# Computational analysis of viable parameter regions in models of synthetic biological systems

**DOI:** 10.1186/s13036-019-0205-0

**Published:** 2019-09-18

**Authors:** žiga Pušnik, Miha Mraz, Nikolaj Zimic, Miha Moškon

**Affiliations:** University of Ljubljana, Faculty of Computer and Information Science, Večna pot 113, Ljubljana, 1000 Slovenia

**Keywords:** Biological model, Repressilator, AC-DC circuit, Biological D flip-flop, Computational analysis, Genetic algorithms, Principal components, Viable parameter regions, Robustness

## Abstract

**Background:**

Gene regulatory networks with different topological and/or dynamical properties might exhibit similar behavior. System that is less perceptive for the perturbations of its internal and external factors should be preferred. Methods for sensitivity and robustness assessment have already been developed and can be roughly divided into local and global approaches. Local methods focus only on the local area around nominal parameter values. This can be problematic when parameters exhibits the desired behavior over a large range of parameter perturbations or when parameter values are unknown. Global methods, on the other hand, investigate the whole space of parameter values and mostly rely on different sampling techniques. This can be computationally inefficient. To address these shortcomings ’glocal’ approaches were developed that apply global and local approaches in an effective and rigorous manner.

**Results:**

Herein, we present a computational approach for ’glocal’ analysis of viable parameter regions in biological models. The methodology is based on the exploration of high-dimensional viable parameter spaces with global and local sampling, clustering and dimensionality reduction techniques. The proposed methodology allows us to efficiently investigate the viable parameter space regions, evaluate the regions which exhibit the largest robustness, and to gather new insights regarding the size and connectivity of the viable parameter regions. We evaluate the proposed methodology on three different synthetic gene regulatory network models, i.e. the repressilator model, the model of the AC-DC circuit and the model of the edge-triggered master-slave D flip-flop.

**Conclusions:**

The proposed methodology provides a rigorous assessment of the shape and size of viable parameter regions based on (1) the mathematical description of the biological system of interest, (2) constraints that define feasible parameter regions and (3) cost function that defines the desired or observed behavior of the system. These insights can be used to assess the robustness of biological systems, even in the case when parameter values are unknown and more importantly, even when there are multiple poorly connected viable parameter regions in the solution space. Moreover, the methodology can be efficiently applied to the analysis of biological systems that exhibit multiple modes of the targeted behavior.

## Background

Biological oscillators govern various biological processes, such as cellular respiration, cardiac functions, and circadian rhythms [1–3]. In the terms of synthetic biology, the research of oscillatory systems is motivated by (1) a better understanding of known biological systems [2, 4–6], and (2) by the development of systems that could potentially be used in practical applications [7, 8]. An implementation of the first synthetic repressilator by Elowitz and Leibler [4] was, together with the synthetic toggle switch by Gardner et al. [9], an important breakthrough in synthetic biology. Since then the focus has shifted from simpler to more complex biological systems [10]. For this reason, the development of robust and *fast-response* systems is of vital importance. For example, Fink et al. [7] designed an artificial fast-response system in mammalian cells that can respond to chemical signals in minutes rather than hours. In the terms of optimization, this can be translated to *multiobjective optimization* [11]. Otero-Muras and Banga [12] recently proposed a multiobjective optimization framework for synthetic biology based on the Pareto optimality. This framework is however limited to the library of synthetic parts, which can be a potential limitation. Moreover, when designing complex biological systems, the desired modes of behavior should not be the only criteria. One should also take into an account the system’s *robustness*, i.e. its stability in terms of correct behavior for a large range of different perturbations of extrinsic and intrinsic factors. If two systems exhibit the same required dynamics, then the more robust system should be preferred [13]. This allows for the development of more efficient and stable biological systems. In order to determine the robustness of the system, one must be able to efficiently explore and characterize its parameter space, for which mathematical modeling is usually applied [14]. To find the optimal parameters that exhibit the desired behavior, different heuristic approaches, such as genetic algorithms (GAs) can be used [14–16]. While GAs have numerous applications, they usually provide only a single near-optimal solution. However, these approaches do not give us an insight into the shape of the solution space and the robustness of the acquired solution. Other approaches are focused on the efficient investigation of the whole parameter regions for which the system displays some predefined behavior. These regions define so-called viable parameter space. Identification of the viable parameter space allows for a more thorough analysis in the context of system’s robustness, sensitivity and possible modes of behavior [13, 17, 18]. Schillings et al. [18] used adaptive Smolyak interpolation that relies on sparse polynomial approximations to characterize the solution space of biochemical networks. The assumption here is that the function we want to interpolate is sufficiently smooth, which is not always the case. This problem can to some extent be addressed with the adaptive interpolation. Li et al. [19] introduced structural and correlative sensitivity analysis (SCSA) which belongs to the family of global sensitivity methods based on the decomposition of variance. Since the viable parameter spaces only represent a small fraction of all feasible solutions, we are more interested in viable regions, and not on the solution space as a whole. Hafner et al. [13] developed a ’glocal’ robustness analysis and model discrimination method that can be used for the analysis of circadian as well as other oscillators. This approach allows us to efficiently explore the model’s parameter space and assess its robustness. One of its major drawbacks is that it is not applicable to biological systems with high dimensional and poorly connected viable parameter regions. Two regions are poorly connected if one cannot traverse the solution space from one viable region to the other with the arbitrarily small steps, while constantly preserving the viability of the current solution. This is not problematic for the evolutionary developed systems, where the viable solution space is usually connected because natural systems have evolved through small, gradual changes of individual biochemical parameters. And while this may be true for the naturally occurring motifs, it is not necessarily the case for the synthetically developed gene regulatory networks (GRNs). When designing synthetic GRNs, one could choose different parts, e.g., transcription factors (TFs) with similar behavior and different kinetic properties, such as binding-site affinities and degradation rates. This problem was also addressed by Zamora-Sillero et al. in [17], where they proposed an efficient ellipsoid based sampling. The limitation of this approach is that the increasing dimensionality may exponentially increase the number of iterations needed to identify all viable parameter regions. This can occur if the viable solution space is loosely connected.

Herein, we present an improved ’glocal’ approach for the computational analysis of viable parameter spaces in high-dimensional dynamical models of biological systems. The methodology is based on the robustness estimation and model analysis methodology described by Hafner et al. [13] and is due to exhaustive sampling with GAs and clustering not only limited to models with connected solution spaces. The methodology consists of multiple steps, i.e. (1) the estimation of viable parameter regions with GAs, (2) efficient exploration of viable regions with local sampling, and (3) robustness estimation for each of the viable regions. Our methodology differs from the one introduced by Hafner et al. [13] in two main aspects. Firstly, we employ GAs for the initial estimation of viable parameter regions, whereas Hafner et al. relies on the literature available data. The problem is that the number of already published viable parameter values can be quite limited for a particular system, and can guide the exploration in the wrong direction. The second important difference is that our approach can account for and discriminate between multiple poorly connected viable regions, whereas the ’glocal’ methodology by Hafner et al. is limited to a single viable region. Moreover, our methodology can be applied to the systems that exhibit multiple modes of behavior, such as alternative current (AC)-direct current (DC) circuit [20]. We evaluate the proposed methodology on the repressilator model, on the model of the AC-DC circuit that can switch between the oscillatory and bistable behavior as described in [20] and on the model of the biological edge-triggered D flip-flop in a master-slave configuration proposed by Magdevska et al. [14]. We perform the analysis of viable regions of the repressilator model with different cost functions. We analyze four different versions of the D flip-flop model, which differ in the functional forms describing the protein degradation (Michaelian versus linear functions) and transcription factor binding at promoter level (competitive versus independent). We validate the results obtained with deterministic simulations with the additional stochastic simulations. These are performed on the randomly selected samples from the viable regions of each model. The proposed methodology is efficient and thorough, and can be applied for the *model-to-model* comparison in terms of their robustness. Finally, it is not limited only to systems that exhibit oscillatory dynamics, but can be applied to complex biological systems with arbitrary dynamics.

## Methods

**Fig. 1:**
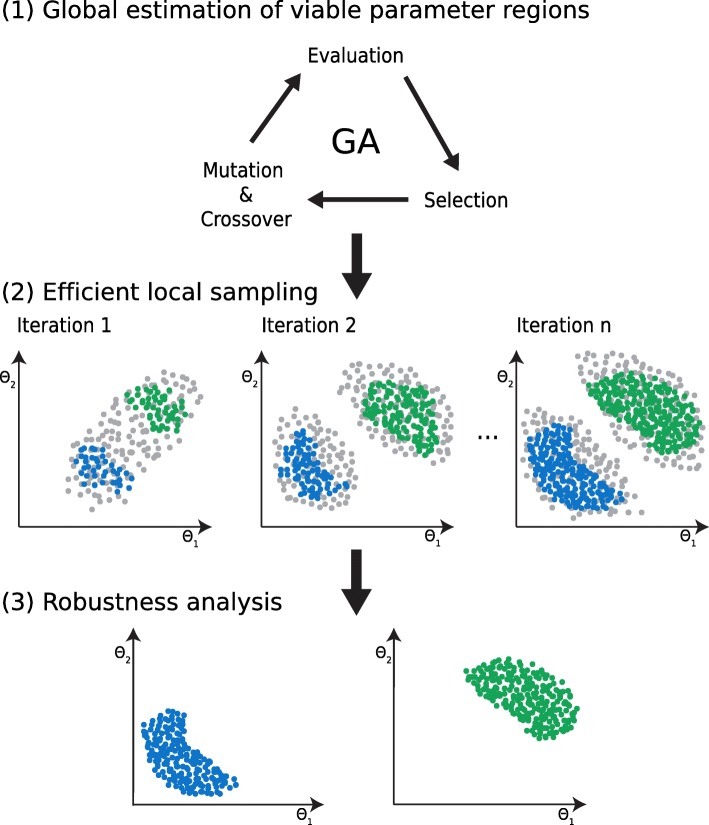
Visualization of the proposed methodology. The methodology is depicted in the following consecutive steps (1) global estimation of viable regions with GA, (2) efficient exploration of viable regions with local sampling, and (3) robustness analysis for the discovered viable regions

**Fig. 2:**
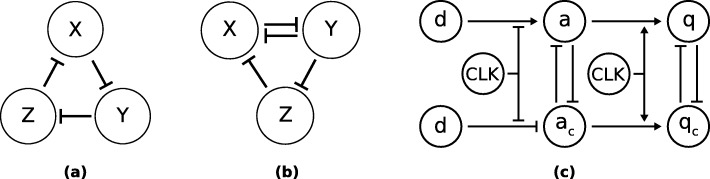
The schematics of biological repressilator, AC-DC circuit and proposed D flip-flop in a master-slave configuration. Figure (**a**) displays the schematic diagram of a repressilator. Proteins *X*, *Y* and *Z* in a negative feedback loop inhibit adjacent proteins. Figure (**b**) displays the design of AC-DC circuit. Mutual inhibition of *X* and *Y* can produce bistable behavior. Figure (**c**) displays the schematic of proposed biological D flip-flop in a master-slave configuration, where *CLK* is the synchronization signal and *d* is the input. Proteins *a* and *a*_*c*_ represent the master segment of the flip-flop, whereas *q* and *q*_*c*_ represent the slave segment

The proposed approach consists of multiple consecutive steps, namely (1) global estimation of viable parameter regions, (2) efficient local sampling, and (3) robustness analysis (see Fig. 1). We apply our approach on models of repressilator, AC-DC circuit, and D flip-flop in a master-slave configuration. Topologies of these models are displayed in Fig. 2.

### Global estimation of viable parameter regions

Nominal parameter values can be extracted from the literature. We are more interested in the analysis of the whole solution space, rather than a single viable solution. Furthermore, in general, nominal values are often unknown or only partially known. The viable parameter space is composed of parameter regions in solution space for which the system exhibits a predefined behavior. More formally, the parameter space can be defined as a Cartesian product

1 $$  E(\theta) = \sum\limits_{i=1}^{n} ({{\hat{h}}_{i} - h_{i}})^{2},  $$

where *Θ*^*p*^ represents the whole parameter space, *p* is the number of parameters and *Θ*_*i*_ represents a range of feasible parameter values for the *i*-th parameter. Ranges of biochemical parameters used in our examples are shown in Table 1. Each point in the parameter space represents a candidate for a viable solution. A parameter point *θ* is viable *iff* the given condition is met

2 $$ E(\theta) < E_{0},  $$

**Table 1:** Possible ranges of parameter values we used in our models

Parameter	Span	Unit
Transcription	10^−2^ - 50	*h* ^−1^
Translation	10^−2^ - 50	*h* ^−1^
Protein production	10^−1^ - 50	*h* ^−1^
Protein degradation	10^−3^ - 50	*h* ^−1^
mRNA degradation	10^−1^ - 100	*h* ^−1^
Dissociation constant	10^−2^ - 250	*nM*
Michaelis constant	10^−2^ - 250	*nM*
Protease concentration	10 - 1000	*nM*
Dilution rate	0.6	*h* ^−1^
Hill coefficient	1 - 5	-
Reaction volume (*V*_*R*_·*N*_*A*_)	1	*n* *M* ^−1^

Values obtained from [40, 45, 46] are a rough estimate of real kinetic rates for prokaryotes and eukaryotes combined. Nevertheless, we excluded the possibility of some extreme cases, like extremely long-lived or unstable proteins. *V*_*R*_ describes reaction space volume and *N*_*A*_ Avogadro constant.

where *E*(*θ*) is a cost value of candidate point and *E*_0_ is a predefined threshold, that divides candidates based on their qualitative behavior, e.g., does a candidate exhibit oscillatory behavior or not. The cost function must be designed in a way to promote candidates that exhibit the desired behavior and in some cases to promote additional biological criteria such as biomass maximization. For edge-triggered D flip-flop in a master-slave configuration, we defined a cost function in the frequency domain as a mean squared error (MSE) between an ideal and the observed signal of the biological system

3 $$  E(\theta) = \sum\limits_{i=1}^{n} ({{\hat{h}}_{i} - h_{i}})^{2},  $$

where *n* is the maximal number of considered harmonics of observed response for candidate *θ*, and $ \hat {h}_{i}, h_{i} $ are *i*-th harmonics of the observed and ideal response, respectively. We have chosen the frequency domain since it better describes the signal in the term of its harmonics and thus simplifies the analysis. An additional useful property of this cost function is its invariance to the phase of the oscillating signal. Nonetheless, this cost function is appropriate only when we are able to define the exact dynamics we would like to achieve for the response of a model. If we are interested in the system behavior in a more qualitative way, e.g., does a system exhibits stable oscillations or not, then the cost function should be defined to cover all possible configurations that dictate the correct behavior. For this reason, we defined the second cost function in the frequency domain, which optimizes the difference between the neighboring peaks and a peak prominence of a response signal in a frequency domain

4 $$  E(\theta) = - \frac{1}{P}\sum_{i=1}^{P}\sigma_{i} - \sum_{i=2}^{P} \left(\gamma_{i} - \gamma_{i-1}\right),  $$

where *γ*_*i*_ is the *i*-th peak, *P* is the number of peaks, and *σ*_*i*_ is standard deviation for the *i*-th peak in the neighboring window of size 3. Ideal sine signals have only one prominent peak. To avoid rewarding the signals with many peaks, we consider only average standard deviation per peak. The minimal size 3 for the window was chosen in order to promote high and narrow peaks. This cost function is defined more loosely in order to not overlook the large range of possible rigid oscillating signals regardless of their amplitude and period, while still favoring undamped oscillations with high amplitudes and a stable periods. We applied both cost functions to the repressilator model to analyze the size and shape of its viable parameter regions.

In the first step of the proposed methodology, the viable regions are estimated by the GA. GAs are inspired by natural evolution and are often used to solve hard optimization problems [15]. Subjects within the population gradually evolve by the means of genetic operators, i.e. mutation, reproduction, and selection. Our initial population consisted of 5000 randomly generated candidates. Each candidate *θ* was represented as a vector of biochemical parameters mutated with predefined probability. In the literature mutation probabilities most often range on the order of 0.01 per position. While this is much higher than in biology, mutation rates should be chosen on the properties and the difficulty of the problem we are aiming to solve [21]. In our case, every biochemical parameter was multiplied with a random value between 0.8 and 1.2 with the probability 0.75 (high mutation probability was set in order to promote greater exploration of solution space). Every parameter can, therefore, increase or decrease in each iteration of GA. Reproduction was implemented using the two-point crossover. Unlike mutation, which serves as a fine-tuning mechanism, the crossover introduces a certain amount of variability in the population, which makes the problem less susceptible to local extrema. At the end of every iteration of genetic algorithm, subjects are evaluated with the appropriate cost function, and only the fraction of the individuals are chosen for the next generation by tournament selection. We used the tournament size of a tenth of the entire population. In contrary to the traditional use of GAs, we sampled all viable subjects, from which the initial viable set *ν*^(0)^ is composed. The exploration of solution space stops when the maximal number of generations is reached. To obtain only the approximate estimation of viable regions, the total number of generations should not be too high. We terminated our GA after 10 generations.

### Efficient local sampling

Since the viable parameter space exploration with GAs is biased towards the *evolvability* of the problem defined with the selection of a cost function, GAs are not appropriate for the final estimation of the solution space. To obtain a more accurate estimate of the viable regions, the solution space is efficiently and thoroughly explored in an iterative manner as described by Hafner et al. [13]. We apply the Gaussian sampling in the direction of the principal components of the explored solution space

5 $$  S^{(i)} = \left\{ E[ \nu^{(i - 1)} ] + \lambda^{(i-1)} \xi_{j} | j = 1,..., N\right\},  $$

where *S*^(*i*)^ is a set composed of candidates for viable solutions in *i*-th iteration of size *N* (in our case *N*=10^5^), *E*[*ν*^(*i*−1)^] is a mean of the viable candidate solutions in the set *ν*^(*i*−1)^ obtained from the previous iteration of the sampling process, *ξ*_*j*_ is the *j*-th Gaussian sample along the principal components of viable set *ν*^(*i*−1)^, and *λ*^(*i*−1)^ is the variance scaling factor. Note that the scaled variance should always be greater than the initial variance of principal components in order to cover the whole viable solution space. We set the initial value of *λ*^(0)^ to 4 and decreased it linearly to 2 in the last, in our case tenth iteration. In this way we focus only on the potential solution areas and avoid unnecessary sampling. At the end of every iteration new viable set *ν*^(*i*)^ is obtained by evaluating candidates in set *S*^(*i*)^. For more details please refer to [13].

To distinguish between poorly connected regions we used the K-means clustering algorithm. This allows for more efficient and accurate characterization of loosely connected viable regions. Since the K-means algorithm expects the number of means, i.e. clusters, one must estimate the correct number of clusters the data is divided into. We tackled this problem with the gap statistic [22]. Let us define *W*_*k*_ as the within-cluster sum of squares around the cluster means. Gap statistic is a cluster analysis algorithm that compares *log*(*W*_*k*_) with its expectation under the appropriate reference null distribution of the data *E*^∗^[*log*(*W*_*k*_)]

6 $$ G(k) = E^{*}[log(W_{k})] - log(W_{k}).  $$

For example consider clustering *n* uniformly distributed points in *p* dimensions. Expected value of *log*(*W*_*k*_) is then

7 $$ E^{*}\left[log(W_{k})\right] = log(pn/12) - (2/p)log(k) + A,  $$

where *k* is a number of clusters and *A* is a constant. If in reality our data consists of *K* well separated clusters, then the *log*(*W*_*k*_) will decrease faster than its expected rate *E*^∗^[*log*(*W*_*k*_)] for *k*≤*K* and slower for *k*>*K*. Hence, when *k*=*K* the gap will be the largest. Similarly, we set the number of clusters in our data according to the largest gap obtained by gap statistic. In [22], the authors proposed two different choices for the reference distribution, namely (1) generate each reference feature uniformly over the range of the observed values, and (2) generate the reference features from a uniform distribution over a bounding hyper-box *B* aligned with the principal components of the data. We have chosen the second approach since it is invariant to the rotation of the data. Note that the same technique was used by Hafner et al. [13] for the purposes of Monte Carlo integration. For more information about gap statistic see [22].

When a viable area is recognized as a separate region, it is extensively explored regardless of any other regions by an iterative procedure described in Eq. (5). This, however, poses a threat that explored viable regions overlap, which makes it harder for *model-to-model* comparison. Alternatively, we can compare only the most robusts regions of both models or combine all regions into a single set.

Due to its computational complexity, clustering is applied only when one of the three criteria is met, namely (1) maximal number of iterations is reached, (2) the number of viable points in next iteration is *n*-times smaller than number of points in the previous iteration (in our case *n*=10), or (3) the convergence of a region *C* exceeds a predefined threshold *C*_0_. Our motivation behind point (2) is that if samples are truly well separated, then the number of viable samples obtained by local sampling will be significantly smaller if we sample points as a single region as opposed to separate sampling for every viable region. Regarding the third point, we defined convergence for region *j* as the Frobenius norm of the difference between current iteration principal components *PC*(*ν*_*j*_)^(*i*)^ and the previous iteration principal components *PC*(*ν*_*j*_)^(*i*−1)^. The reason for the selection of this norm is that the main principal components directly influence the direction of sampling in consecutive iterations. We thus defined convergence as

8 $$ C_{j}^{(i)} = \left|\left| PC(\nu_{j})^{(i)} - PC(\nu_{j})^{(i-1)} \right|\right|_{F}.  $$

This allows us to avoid unnecessary clustering at the beginning of the sampling process when the number of viable candidates does not suffice for representative clustering results. This iterative process stops when all regions are sufficiently explored, i.e. principal components do not change over next iterations or the maximal number of iterations is reached.

### Robustness analysis

A model of the robust system can cope with a large range of changes of internal or external factors, e.g., temperature, that can directly influence its kinetic parameters. The more robust model will in general have a greater volume of viable parameter regions and will be able to cope with perturbations of its parameters. To assess the model robustness, we estimate the volume of each viable region *V* with Monte Carlo integration [13, 23, 24]

9 $$ \textit{Vol} (V) \approx \left(|\nu|/|S|\right)*\textit{Vol}(B),  $$

where *Vol*(*B*) is the volume of a bounding hyper-box *B* aligned with principal components of the data, |*S*| is the number of uniformly generated samples within *B* and |*ν*| is the number of viable samples within *S*. Because principal components are perpendicular, the volume of *B* can be calculated as a product across lengths of hyper-box edges. Sampling within *B* therefore drastically reduces the number of samples required to estimate the viable volume in the same precision as opposed to uniform sampling of the whole parameter space. Note that instead of bounding box one could also calculate the convex hull and calculate its volume. This is computationally more expensive compared to the bounding box approach, especially when considering that we already calculated the principal components in the previous step of our methodology. To make *model-to-model* comparison we cannot disregard that models can have a different number of dimensions. For this reason, we normalized the viable volume to obtain a relative volume, which we calculated as a ratio between the viable volume and the volume of the total solution space

10 $$ \textit{Vol}~^{\prime}(V) = \textit{Vol}(V) \mathbin{/} \textit{Vol}(\Theta^{p}).  $$

One question remains. What is the required number of samples in Monte Carlo simulations to assess the viable volume within the error *δ* for a confidence *β*? Every uniformly generated sample *θ* within the bounding box *B* can be viewed as a Bernoulli random variable

11 $$ U = \begin{cases} 1 & \text{if \(\theta\) is viable,}\\ 0 & \text{otherwise.}\\ \end{cases}  $$

The mean of *U* is then $\mu = \frac {\textit {Vol}(V)}{\textit {Vol}(B)}$. The standard deviation *σ* is not known. Since *U* is a Bernoulli random variable, the maximal variance *σ*^2^ is 1/4. The central limit theorem can then be applied

12 $$\begin{array}{@{}rcl@{}} P\left(\left| \frac{|\nu|}{|S|}*\textit{Vol}(B) - \textit{Vol}(V) \right| \leq \delta \right) =  \\ P\left(\left| \frac{|\nu|}{|S|} - \mu \right| \leq \frac{\delta}{\textit{Vol}(B)} \right) \approx  \\ P\left(|Z| \leq \frac{\delta* \sqrt{|S|}}{\sigma*\textit{Vol}(B)} \right) \approx  \\ 2P\left(Z \leq \frac{2\delta* \sqrt{|S|}}{\textit{Vol}(B)} \right) - 1, \end{array} $$

where *Z* is a normally distributed random variable with zero mean and unit variance. To get $2P\left (Z \leq \frac {2\delta * \sqrt {|S|}}{\textit {Vol}(B)} \right) - 1 \geq \beta $, the number of samples |*S*| must be at least

13 $$ |S| \geq \left| \frac{\Phi^{-1}\left(\frac{\beta - 1}{2}\right)*\textit{Vol}(B)}{2\delta} \right|^{2},  $$

where *Φ*^−1^ is inverse cumulative distribution function of *Z*. For a confidence level of 0.95, we get $|S| \geq \left | \frac {0.98 * \textit {Vol}(B)}{\delta } \right |^{2}$. In other words, if we want to assess the viable volume within one percent of the total bounding box volume with the confidence level of 0.95, *S* should contain at least 10^4^ samples.

## Results

### Repressilator

**Fig. 3:**
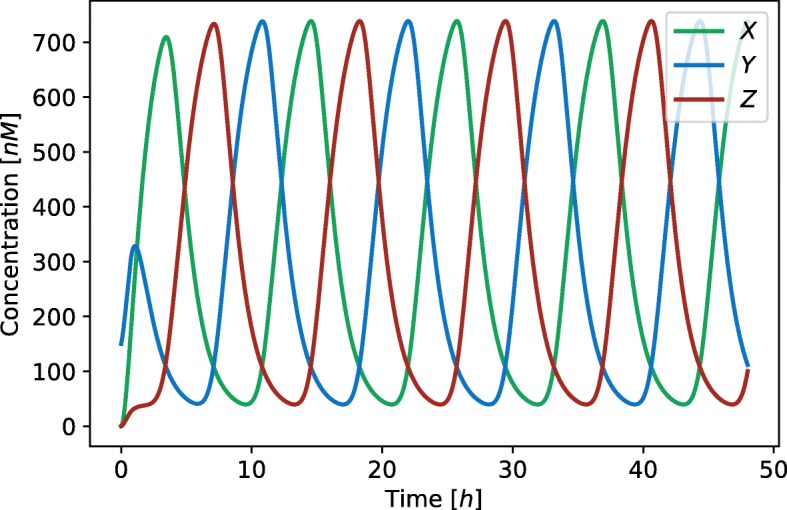
Results of a deterministic simulation performed on the repressilator model. Blue, red and green represent the concentration of proteins *X*, *Y* and *Z*, respectively. The duration of simulation is 48 *h* with the initial conditions *X*=150*nM*, *Y*=0*nM*, *Z*=0*nM*, and *α*=49.61*h*^−1^, *α*_0_=1.43*h*^−1^, *n*=4.4, *β*=21.83*h*^−1^, *δ*_*m*_=1.72*h*^−1^, *δ*_*p*_=0.78*h*^−1^ and *Kd*=123.12*nM*. Protein concentration oscillate with the period of 12 *h* and the amplitude of 300 *nM*

Repressilator is a simple GRN composed of an odd number of repressors connected in a negative feedback loop. The simplest repressilator is a Goodwin oscillator, which consists of a single repressor [25]. We are interested in the repressilator with three repressors, first described by Elowitz and Leibler [4]. The GRN topology of the repressilator is displayed in Fig. 2 (a). The dynamics of repressilator can be described by the following ordinary differential equations (ODEs)

14 $$\begin{array}{@{}rcl@{}} \frac{dmX}{dt} =& -\delta_{m} mX + \frac{\alpha}{1 + \left(\frac{Z}{Kd}\right)^{n}} + \alpha_{0}, \end{array} $$

15 $$\begin{array}{@{}rcl@{}} \frac{dmY}{dt} =& -\delta_{m} mY + \frac{\alpha}{1 + \left(\frac{X}{Kd}\right)^{n}} + \alpha_{0}, \end{array} $$

16 $$\begin{array}{@{}rcl@{}} \frac{dmZ}{dt} =& -\delta_{m} mZ + \frac{\alpha}{1 + \left(\frac{Y}{Kd}\right)^{n}} + \alpha_{0}, \end{array} $$

17 $$\begin{array}{@{}rcl@{}} \frac{dX}{dt} =& \beta mX - \delta_{p} X, \end{array} $$

18 $$\begin{array}{@{}rcl@{}} \frac{dY}{dt} =& \beta mY - \delta_{p} Y, \end{array} $$

19 $$\begin{array}{@{}rcl@{}} \frac{dZ}{dt} =& \beta mZ - \delta_{p} Z, \end{array} $$

where *mX*, *mY* and *mZ* are mRNA concentrations of repressors *X*, *Y* and *Z*, respectively. Parameter *δ*_*m*_ is mRNA degradation rate, *δ*_*p*_ protein degradation rate, *α* transcription rate, *α*_0_ leakage rate, *β* translation rate, and *Kd* dissociation constant. Hill coefficient *n* describes the strength of cooperative binding between transcription factors. The deterministic simulation of repressilator is shown in Fig. 3.

**Fig. 4:**
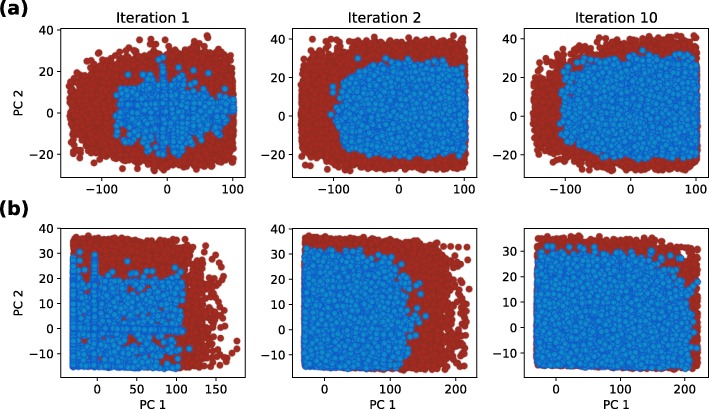
Exploration of viable regions of the repressilator models. Blue dots represent viable solutions, red dots represent candidates for viable solutions. Blue dots in Iteration 1 correspond to the viable solutions obtained with GA. Figure (**a**) displays the exploration of viable regions based on the first cost function, Figure (**b**) displays the exploration of viable regions based on the second, more loosely defined cost function

**Fig. 5:**
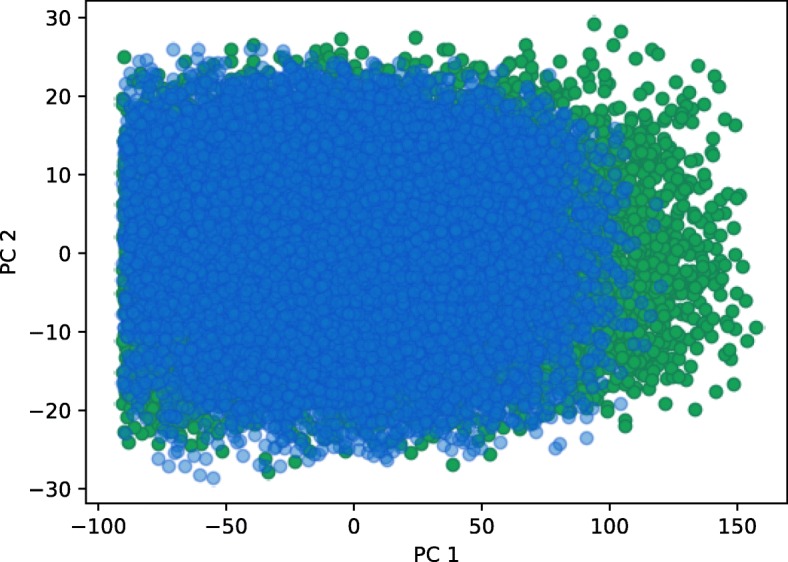
The projection of repressilator viable regions on the two main principal components for different cost functions. Blue dots represent samples obtained with the first cost function (Eq. 3), green dots represent samples obtained with the second cost function (Eq. 4). While both viable regions overlap, the viable region obtained with the first cost function is clearly smaller

**Fig. 6:**
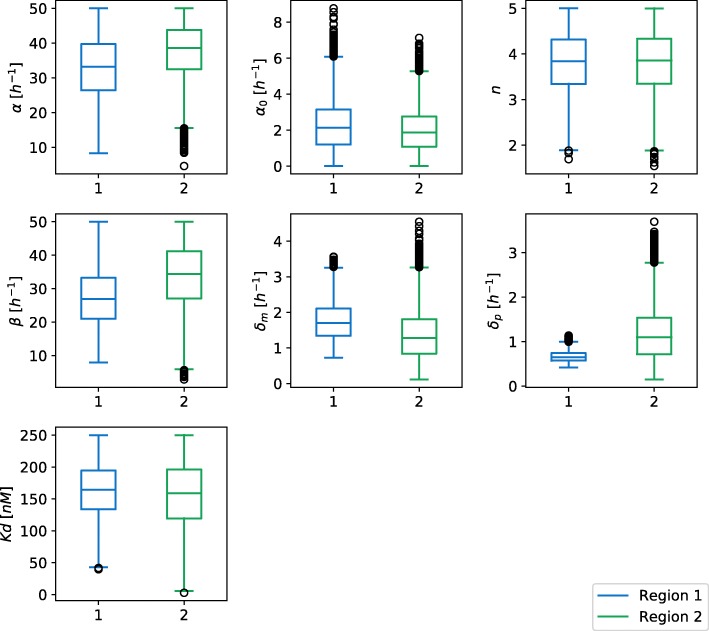
Boxplots of viable parameter regions for the deterministic model of repressilator. The parameters that we observed are *α*, *α*_0_, *β*, *δ*_*m*_, *δ*_*p*_, *n* and *Kd*. We can see that the parameters span approximately over the same range of values, except the parameter *δ*_*p*_, which has significantly smaller range for viable region 1 compared to the viable region 2. Viable region 1 corresponds to scenario 1, region 2 corresponds to scenario 2

To analyze the effect of selecting different cost function on the size and shape of viable parameter regions, we studied two different scenarios. In the first scenario we applied cost function from the Eq. (3) to obtain a signal with amplitude of 300 *nM* and a period of 12 *h*. We considered a parameter point viable if the harmonics of its response did not deviate, on average, for more than 10 *nM* from the harmonics of the ideal signal. The first, second and last iteration of exploration of viable regions for scenario 1 are displayed in Fig. 4 (a). Parameter space exploration is projected to the first two principal components. We can observe that the viable space is well defined and consists of a single region, which is also confirmed by a gap statistic that predicted one optimal cluster. Our results are consistent with the findings of other researches [13, 17]. For the second scenario, we used Eq. (4) as a cost function and considered parameter point viable if the amplitude of its response was roughly between 200 *nM* and 400 *nM*, and its cost value below −200. We estimated the amplitude of the signal with the size of its leading harmonic. The exploration of viable regions for the second scenario is displayed in Fig. 4 (b). Here, similarly to the first scenario, the solution space is well defined and connected. The obtained solution spaces can be directly compared on the basis of their volume. The viable volume for scenario 2 is by our observations approximately 20-fold larger than for scenario 1. Since the second scenario has a more loosely defined cost function, this coincides with our expectations. We can compare the volumes in Fig. 5, in which we projected two viable regions to their main principal components. Figure 6 represents the boxplots of parameter values for both cases. We can observe that the range of values the parameters span, is in the same range for both regions, except for the parameter *δ*_*p*_. In the second scenario, *δ*_*p*_ has significantly greater span compared to the first one, which is also the main reason behind the difference in the viable volumes.

**Fig. 7:**
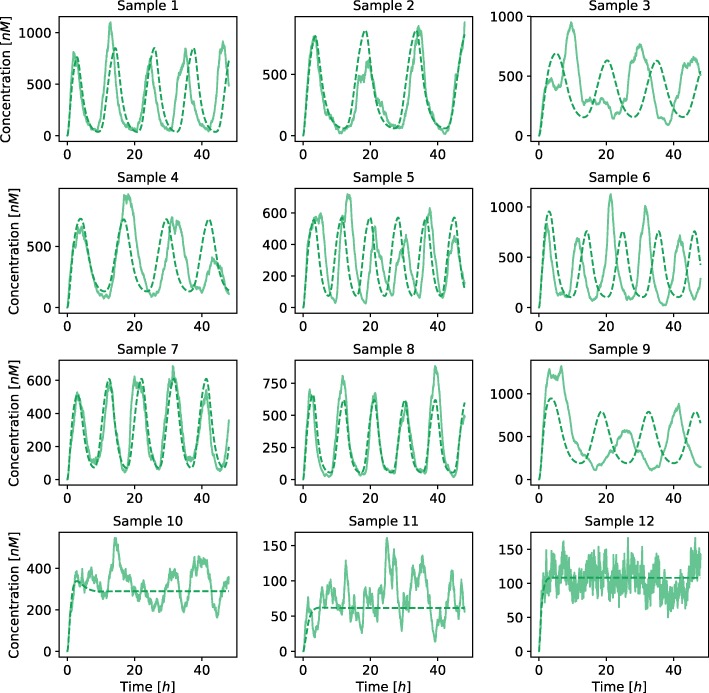
Comparison of stochastic and deterministic simulations performed on the repressilator model. In the first three rows the parameters were randomly sampled from the viable solution space. The last row presents the results of the simulations where the parameters were sampled outside the viable solution space. Solid lines present the results of stochastic simulations and dashed lines present the results of deterministic simulations. Each of the lines presents the evolution of protein *X* concentrations

**Fig. 8:**
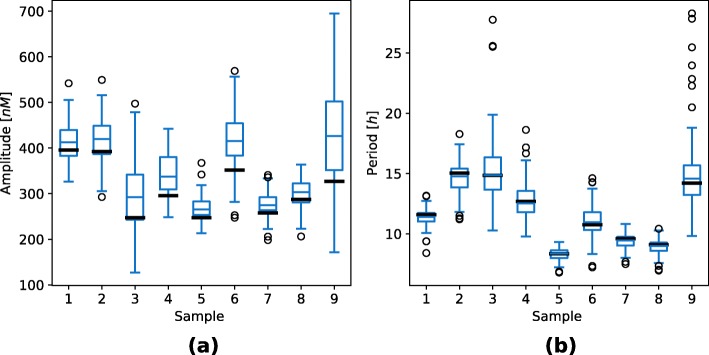
Boxplots visualizing the repressilator amplitudes and periods obtained with stochastic simulations. Figure (**a**) presents the average oscillation amplitudes and Figure (**b**) the average oscillation periods. Sample numbers correspond to the numbers used in Fig. 7. Only viable samples are displayed here. A hundred simulations were performed for each sample. Average oscillation amplitudes and periods were measured in each of the simulations. Bold black lines present the oscillation amplitudes and periods observed in deterministic simulations

Deterministic simulation approaches are only able to describe average response of the system and do not directly account for the noise influences. We additionally validated the obtained results with the execution of stochastic simulations using stochastic simulation algorithm (SSA) [26, 27] and its quasi steady-state approximation (QSSA) [28]. Latter allowed us to directly project the deterministic reaction system to its stochastic equivalent. We randomly selected nine points from the viable solution space and three points outside the viable solution space to compare the results of stochastic and deterministic simulations (see Fig. 7). We repeated each stochastic simulation for a hundred times and measured the average amplitudes and periods, which are visualized in Fig. 8. Points sampled from the feasible parameter regions pertained the oscillatory behavior also in the stochastic simulations. Moreover, periods were within the same ranges as in the deterministic simulations. Larger amplitudes were observed in stochastic simulation results, presumably due to intrinsic noise, which is not regarded in deterministic simulations. Points which reflected stationary behavior in deterministic simulations pertained this dynamics in stochastic simulations as well.

### AC-DC circuit exhibits bistability and oscillations

**Fig. 9:**
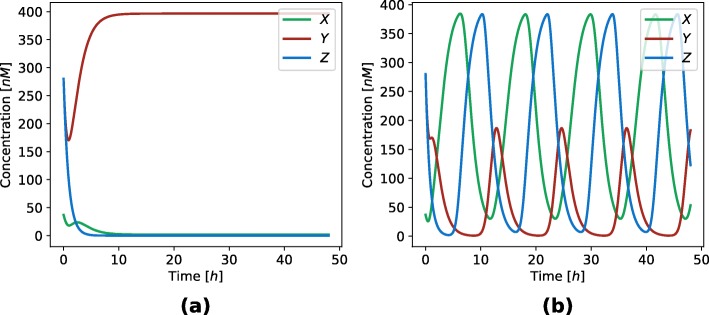
Results of a deterministic simulations performed on the AC-DC circuit model. Blue, red and green represent the concentration of proteins *X*, *Y* and *Z*, respectively. The duration of simulation is 48 *h* with the initial conditions *X*=37*nM*, *Y*=280*nM*, *Z*=280*nM*. Figure (**a**) shows the bistable dynamics for a point in the parameter space *α*=5.91*h*^−1^, *n*=4.16, *β*=47.35*h*^−1^, *δ*_*m*_=0.63*h*^−1^, *δ*_*p*_=1.12*h*^−1^, *Kd*_*a*_=165.03*nM*, *Kd*_*b*_=111.2*nM*, *Kd*_*c*_=101.5*nM*, *Kd*_*d*_=0.48*nM*. The initial concentration of protein *X* is low and *Y* is high. Through time the concentrations of proteins *X* and *Y* balanced at 0 and 400 *nM*, respectively. Figure (**b**) shows the oscillating dynamics for the parameter point *α*=26.26*h*^−1^, *n*=3.82, *β*=18.14*h*^−1^, *δ*_*m*_=0.92*h*^−1^, *δ*_*p*_=1.29*h*^−1^, *Kd*_*a*_=151.42*nM*, *Kd*_*b*_=197.61*nM*, *Kd*_*c*_=41.88*nM*, *Kd*_*d*_=11.7*nM*. Proteins *X* and *Z* have the same amplitude of 200 *nM*, while the amplitude of protein *Y* is around 80 *nM*. All proteins have the same period of 12 hours

The AC-DC circuit described by Panovska-Griffiths et al. [20] presents the combination of the toggle switch and the repressilator circuit. This GRN pattern has a natural role in the determination of the spatial organization of cell type generation and aids in the tissue development of the ventral regions of the vertebrate neural tube [20]. The topology of this circuit allows switch-like as well as oscillatory behavior by changing the concentration of the signaling molecule *S*. Moreover, Perez-Carrasco et al. [29] showed that the coexistence of oscillatory and stable gene expression can in the dependence of the intrinsic noise give rise to the coherence of oscillations. We are interested in the multi-modal behavior of the AC-DC circuit. For the sake of simplicity, we excluded the signaling molecule from the model and focused on two modes of behavior, i.e. bistable and oscillatory behavior. The schematic of the AC-DC circuit is displayed in Fig. 2 (b). The dynamics of the AC-DC circuit can be described by the following equations

20 $$\begin{array}{@{}rcl@{}} \frac{dmX}{dt} =& -\delta_{m} mX + \frac{\alpha}{1 + \left(\frac{Z}{Kd_{a}}\right)^{n} + \left(\frac{Y}{Kd_{b}}\right)^{n}},  \end{array} $$

21 $$\begin{array}{@{}rcl@{}} \frac{dmY}{dt} =& -\delta_{m} mY + \frac{\alpha}{1 + \left(\frac{X}{Kd_{c}}\right)^{n}},  \end{array} $$

22 $$\begin{array}{@{}rcl@{}} \frac{dmZ}{dt} =& -\delta_{m} mZ + \frac{\alpha}{1 + \left(\frac{Y}{Kd_{d}}\right)^{n}}, \end{array} $$

23 $$\begin{array}{@{}rcl@{}} \frac{dX}{dt} =& \beta mX - \delta_{p} X, \end{array} $$

24 $$\begin{array}{@{}rcl@{}} \frac{dY}{dt} =& \beta mY - \delta_{p} Y, \end{array} $$

25 $$\begin{array}{@{}rcl@{}} \frac{dZ}{dt} =& \beta mZ - \delta_{p} Z, \end{array} $$

where *mX*, *mY* and *mZ* are mRNA concentrations of proteins *X*, *Y* and *Z*, respectively. Parameter *δ*_*m*_ is mRNA degradation rate, *δ*_*p*_ protein degradation rate, *α* transcription rate, *β* translation rate, and *n* is a Hill coefficient. *Kd*_*a*_, *Kd*_*b*_, *Kd*_*c*_, *Kd*_*d*_ are dissociation constants. Notice the similarity to the repressilator model, whereas the third and the second term in the denominator of Eqs. (20, 21) correspond to the toggle switch dynamics. The results of deterministic simulations for both modes of behavior are displayed in Fig. 9. The same model can exhibit different modes of behavior with the same initial conditions and different kinetic parameter values.

We regarded a parameter point viable if its response exhibited oscillations or bistability. We tested the bistability with two scenarios. First, the initial concentration of protein *X* was low and *Y* was high and vice versa in the second scenario. Throughout the simulation, the protein with the initial high concentration should stabilize at 400 *nM* and the protein with initial low concentration should stabilize at 0 *nM*. To optimize the bistable behavior we resorted to the cost function (Eq. 3), where we directly compared the response in the time domain instead of in the frequency domain. If the observed response did not deviate, on average, from the ideal signal for more than 4 *nM*, we considered a parameter point viable.

**Fig. 10:**
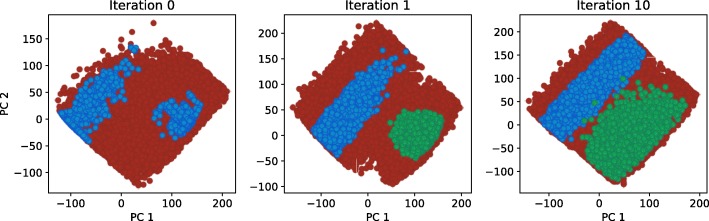
Exploration of viable regions for the AC-DC circuit. Blue and green dots represent viable solutions, red dots represent candidates for viable solutions. Blue dots in Iteration 1 and 10 correspond to viable solutions with bistable dynamics, while green dots correspond to solutions with oscillatory dynamics

Equivalently, we considered a parameter point viable if it exhibited oscillations. We applied the cost function from Eq. (3). We considered only the first ten harmonics and treated a point in parameter space viable if the difference between the harmonics of its response and the ideal response did not exceed 15 *nM* per harmonic on average. We set the amplitude of the ideal signal to 200 *nM* and the period to 12 *h*. We thus defined two cost functions, one for each scenario and ran GA twice. In the local sampling step, we concatenated all viable solutions together and treated every sample from the parameter space equally, regardless of its behavior, i.e. oscillations or bistability. Because the number of viable samples in the first iteration of local sampling was to low (10-times smaller), clustering was performed and two distinct regions were obtained. After that, each region was explored separately for 10 iterations and the exploration was terminated regardless of the gap statistic predictions. Figure 10 displays the course of the exploration of viable regions. Our approach correctly identified both viable regions and explored them independently.

**Fig. 11:**
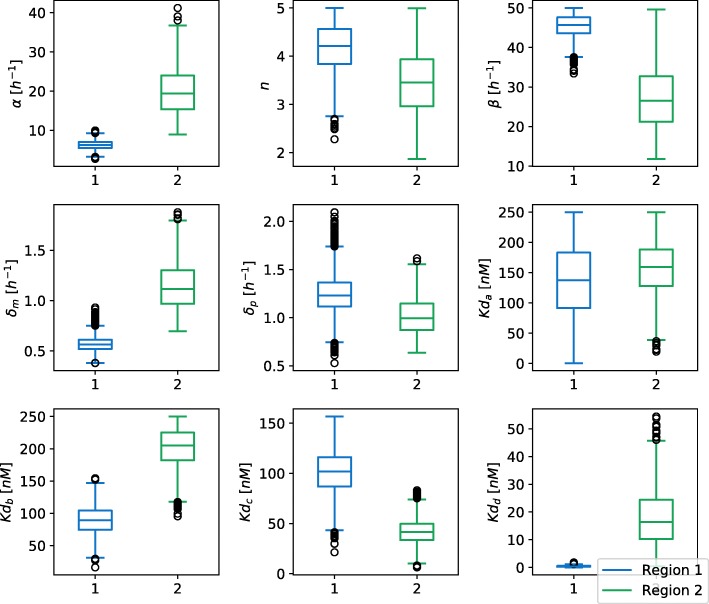
Box plots of values each parameter can span in both viable regions for the deterministic model of AC-DC circuit. The observed parameters are *α*, *n*, *β*, *δ*_*m*_, *δ*_*p*_, *Kd*_*a*_, *Kd*_*b*_, *Kd*_*c*_ and *Kd*_*d*_. We can see that the parameters span generally over different areas for both viable regions. Region 1 is bistable, region 2 oscillates

We also assessed the robustness to the perturbation of parameters for both regions. A viable region with the oscillatory behavior has an approximately 500-fold greater volume that the viable regions with bistable dynamics. This can be confirmed with the boxplots of the parameters spans in both viable regions (see Fig. 11). Transcription (*α*), translation (*β*), mRNA degradation (*δ*_*m*_) and dissociation constant (*Kd*_*d*_) have significantly smaller span for the bistable region than for the region with the oscillatory behavior. Our results indicate that the AC-DC model tends to the oscillations more than to the bistability.

**Fig. 12:**
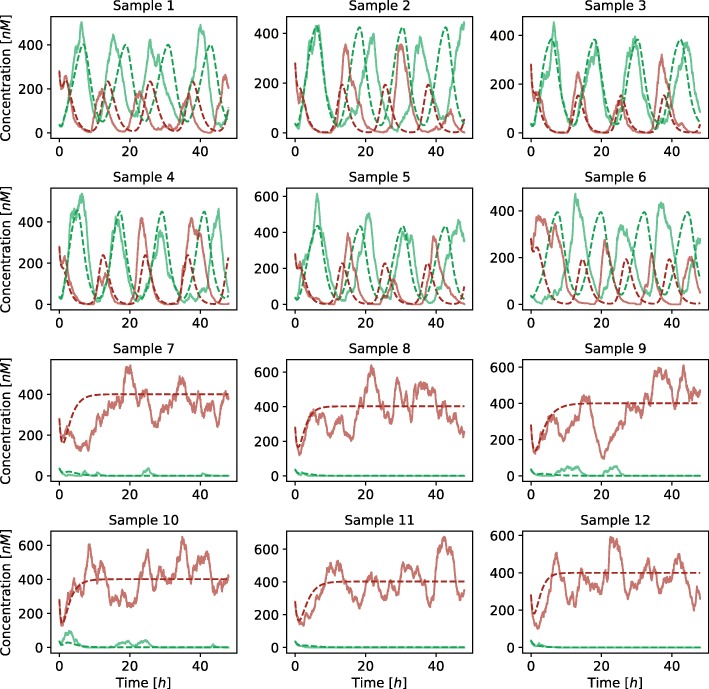
Comparison of stochastic and deterministic simulations performed on the AC-DC circuit model. In the first two rows the parameters were randomly sampled from the viable solution space defining oscillatory behavior (region 2). In the last two rows the parameters were randomly sampled from the viable solution space defining bistable behavior (region 1). Solid lines present the results of stochastic simulations and dashed lines present the results of deterministic simulations. Each of the lines presents the evolution of protein *X* (green) and *Y* (red) concentrations

**Fig. 13:**
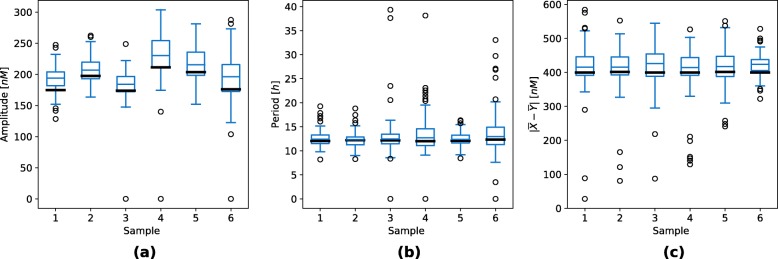
Boxplots visualizing the stochastic response of the AC-DC circuit, namely amplitudes and periods in the oscillatory mode and distance between the stable states in the bistable mode. Figures (**a**) and (**b**) correspond to the oscillatory mode of the circuit. Figure (**a**) presents the average oscillation amplitudes and Figure (**b**) the average oscillation periods. Figure (**c**) corresponds to the bistable mode of the circuit and presents the average distances between the stable states in each of the stochastic simulations. Sample numbers correspond to the numbers used in Figure 12. A hundred simulations were performed for each sample. Average oscillation amplitudes and periods or average distances between the stable states were measured in each of the simulations. Bold black lines present the results observed in deterministic simulations for the corresponding samples

We validated the obtained results with the execution of stochastic simulations in a similar way as in the repressilator model. We randomly selected six random points which reflected oscillatory behavior and six random points which reflected bistable behavior in the deterministic simulations. All of the selected samples reflected the predefined dynamics in stochastic simulations as well (see Fig. 12). We repeated each stochastic simulation for a hundred times and measured the average amplitudes and periods for the samples that reflected oscillatory behavior, and average distance between the stable states for the samples that reflected bistability. The obtained results are visualized in Fig. 13. As in the case of the repressilator model, larger amplitudes were observed in stochastic simulation results, while the periods were approximately the same.

### Edge-triggered d flip-flop in a master-slave configuration

In electronics, D (delay) flip-flop is a memory circuit that exhibits two stable states and can thus store 1 bit of information. D flip-flop delays the input bit for one clock cycle and is triggered by high or low clock levels. The clock signal (CLK) is a synchronization signal frequently used in electronics. The sensitivity on high or low levels of CLK can be problematic when the input signal is not exactly synchronized with CLK, which can result in unpredictable behavior. The master-slave configuration solves this problem by connecting two flip-flops successively. The master flip-flop is triggered on the low CLK levels and the slave flip-flop on the high CLK levels. The D flip-flop in a master-slave configuration is therefore triggered solely on the positive edge of the CLK signal. In biological terms, the CLK represents a synchronization signal that governs periodic cellular processes. A deterministic model of biological edge-triggered D flip-flop based on ODEs was already established and studied by Magdevska et al. [14]. Recently, Andrews et al. [30] demonstrated the implementation of sequential logic in cells with genetic Negated OR (NOR) gates. Among others, they implemented and successfully verified the correct behavior of a gated D flip-flop in *Escherichia coli* (*E. coli*). Their flip-flop correctly switched and maintained states for more than 2 days, and accurately responded to a synchronization signal. Nonetheless, there are two distinctions between their implementation and one proposed by Magdevska et al. Flip-flop by Andrews et al. is constructed with NOR gates, and responds only on the high levels of the synchronization signal, whereas Magdevska et al. proposed the edge-triggered flip-flop, which is composed of two bistable switches regulated with delay, i.e. *d*, and synchronization, i.e. CLK signals. In the latter, when *d*=*q*_*c*_, flip-flop oscillates between a high and low state with twice the period of CLK signal. In this way, flip-flop acts as a 1-bit counter. We used this property to obtain the desired dynamics with GAs. We applied our methodology on the flip-flop topology described in [14], which can be presented with the following ODE-based model

26 $${} {\begin{aligned} \frac{da}{dt} =&\, \alpha_{1}\frac{\left(\frac{d}{Kd}\right)^{n}}{1 + \left(\frac{d}{Kd}\right)^{n} + \left(\frac{CLK}{Kd}\right)^{n} + \Omega_{1}\left(\frac{d}{Kd}\right)^{n}\left(\frac{CLK}{Kd}\right)^{n}} + \alpha_{2}\frac{1}{1 + \left(\frac{a_c}{Kd}\right)^{n}} -\\ &a f_{deg}\left(\Omega_2, \delta_1, \delta_{dil}, E, K_M, a + a_c + q + q_{c}\right) \end{aligned}}  $$

27 $${} {\begin{aligned} \frac{da_{c}}{dt} =&\ \alpha_{1}\frac{1}{1+ \left(\frac{d}{Kd}\right)^n + \left(\frac{CLK}{Kd}\right)^n +\Omega_{1}\left(\frac{d}{Kd}\right)^{n}\left(\frac{CLK}{Kd}\right)^{n}} + \alpha_{2}\frac{1}{1 + \left(\frac{a}{Kd}\right)^{n}} -\\ & a_c f_{deg}\left(\Omega_2, \delta_1, \delta_{dil}, E, K_M, a + a_c + q + q_{c}\right) \end{aligned}}  $$

28 $${} {\begin{aligned} \frac{dq}{dt} =& \alpha_{3}\frac{\left(\frac{a}{Kd}\right)^{n}\left(\frac{CLK}{Kd}\right)^{n}}{1 + \left(\frac{a}{Kd}\right)^{n} + \left(\frac{CLK}{Kd}\right)^{n} + \left(\frac{a}{Kd}\right)^{n}\left(\frac{CLK}{Kd}\right)^{n}} + \alpha_{4}\frac{1}{1 + \left(\frac{q_{c}}{Kd}\right)^{n}} - \\ &q f_{deg}\left(\Omega_{2}, \delta_{2}, \delta_{dil}, E, K_{M}, a + a_{c} + q + q_{c}\right), \end{aligned}}  $$

29 $${} {\begin{aligned} \frac{dq_{c}}{dt} =& \alpha_{3}\frac{\left(\frac{a_{c}}{Kd}\right)^{n}\left(\frac{CLK}{Kd}\right)^{n}}{1 + \left(\frac{a_{c}}{Kd}\right)^{n} + \left(\frac{CLK}{Kd}\right)^{n} + \left(\frac{a_{c}}{Kd}\right)^{n}\left(\frac{CLK}{Kd}\right)^{n}} + \alpha_{4}\frac{1}{1 + (\frac{q}{Kd})^{n}} - \\ &q_{c} f_{deg}\left(\Omega_{2}, \delta_{2}, \delta_{dil}, E, K_{M}, a + a_{c} + q + q_{c}\right),  \end{aligned}}  $$

where

30 $${}  f_{deg}\left(\Omega, \delta, \delta_{dil}, E, K_{M}, P\right)= \left\{ \begin{array}{ll} \frac{\delta E}{K_{M} + P} + \delta_{dil};&\text{\textit{if }} \Omega = 0\\ \delta;&\text{\textit{if }} \Omega = 1. \end{array} \right.  $$

**Fig. 14:**
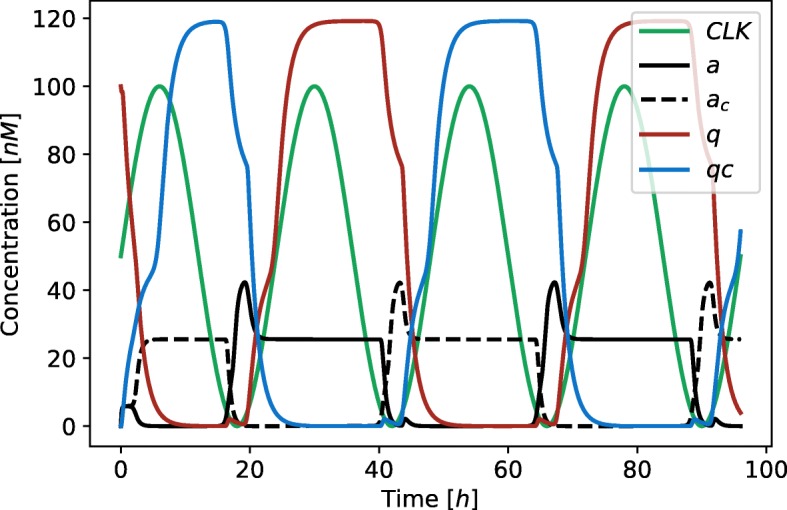
Results of a deterministic simulation performed on the model of the D flip-flop in a master-slave configuration with independent binding of transcription factors (*Ω*_1_=1) and linear degradation term (*Ω*_2_=1). Black, black-dashed, green and blue lines represent the concentration of proteins *a*, *a*_*c*_, *q* and *q*_*c*_, respectively. Green line represent the *CLK* signal with the 24 *h* period. The parameters of the model are *α*_1_=34.73*h*^−1^, *α*_2_=49.36*h*^−1^, *α*_3_=32.73*h*^−1^, *α*_4_=49.54*h*^−1^, *δ*_1_=1.93*h*^−1^, *δ*_2_=0.69*h*^−1^, *Kd*=4.44*nM* and *n*=4.35

Parameters *α*_1_, *α*_2_, *α*_3_ and *α*_4_ are the expression rates of proteins *a*, *a*_*c*_, *q* and *q*_*c*_, *Kd* and *K*_*M*_ are dissociation and Michaelis constants, *n* is Hill coefficient, *δ*_1_, *δ*_2_ are degradation rates of the observed proteins, *δ*_*dil*_ is dilution rate and *E* is the total protease concentration. We introduce parameters *Ω*_1_ and *Ω*_2_ to study the effects of different functional forms describing the binding of transcription factors to the promoters and the effects of different models of protein degradation. Namely, when *Ω*_1_ equals 0 we presume competitive binding of transcription factors to the promoters regulating the expression of *a* and *a*_*c*_. When *Ω*_1_ equals 1 independent binding is presumed. Parameter *Ω*_2_ defines the degradation model for which we use either linear or Michaelian form (see Eq. 30). The Michaelian model of degradation was derived in the same manner as described in [31] and [32]. The schematic of a general biological D flip-flop is shown in Fig. 2 (c). Results of a deterministic simulation for the flip-flop model with independent binding of transcription factors (*Ω*_1_=1) and linear degradation term (*Ω*_2_=1) are shown in Fig. 14.

**Fig. 15:**
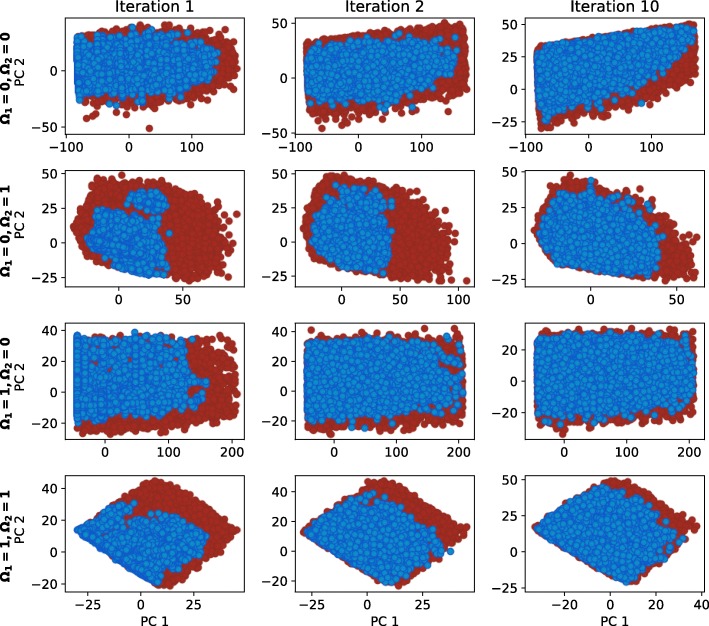
The exploration of viable regions for the edge-triggered D flip-flop. *Ω*_1_ defines competitive (0) or noncompetitive (1) binding sites at promoter level, and *Ω*_2_ Michaelian (0) or linear (1) protein degradation form. Blue dots represent the viable solutions, red dots represent candidates for viable solutions

Parameters *Ω*_1_ and *Ω*_2_ define different functional forms of the same flip-flop model. We were interested in the comparison of the solution spaces for each of the functional forms. We evaluated the viable solution space and its volume for each of the four combinations, i.e competitive versus independent binding and Michaelian versus linear protein degradation. To obtain the viable set of parameters, we adopted the cost function from Eq. (3) and deemed the parameter point non-viable if the difference between the first ten harmonics of its response and the ideal signal exceeded, on average, 10 *nM*s per harmonic. The amplitude of the ideal signal was set to 50 *nM* and the period was set to 48 *h*. Figure 15 presents the exploration of viable regions for each of the four functional form combinations. Our approach identified single well-connected solution space in each case.

**Fig. 16:**
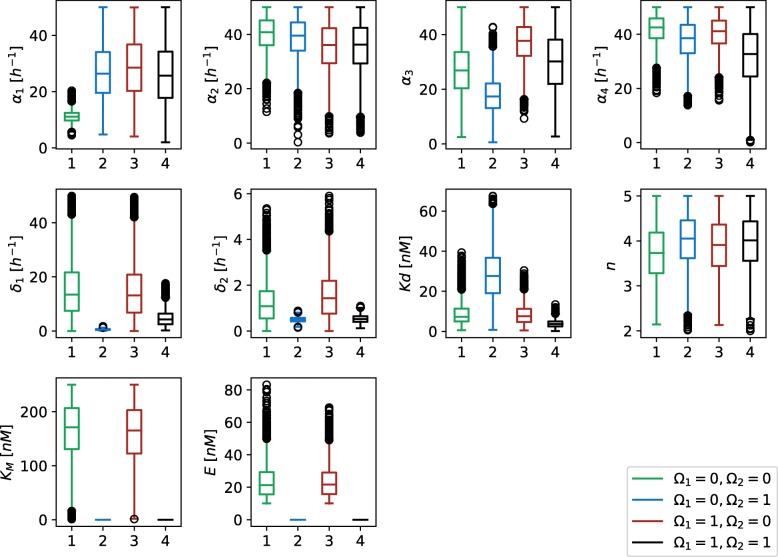
Box plots of values each parameter can span in the viable regions for the deterministic model of D flip-flop. *Ω*_1_ defines competitive (0) or noncompetitive (1) binding sites at promoter level, and *Ω*_2_ Michaelian (0) or linear (1) protein degradation form. Parameters *K*_*M*_ and *E* are not used in the linear protein degradation model

To assess the robustness of different functional form combinations we evaluated the relative volumes of their solution spaces, which are presented in Table 2. The results indicate that Michaelian protein degradation form increases the solutions space and thus the robustness of the proposed topology, but not as much as noncompetitive transcription factor binding at promoter level. We wanted to confirm this hypothesis with the investigation of parameter spans in each of the solution spaces. These are presented in Fig. 16. The spans of protein degradation rates (*δ*_1_ and *δ*_2_) are larger when the Michaelian protein degradation model is presumed. When comparing the models with Michaelian protein degradation form, noncompetitive binding increases or pertains the span of feasible parameter ranges. The same holds for the linear protein degradation form, except in the case of dissociation constant, where larger span is observed for competitive scenario.

**Table 2:** Approximated relative volumes of the feasible solution spaces in the D flip-flop model

*Ω*_1_	*Ω*_2_	Relative volume (*Vol*^′^)
0	0	3.3·10^−6^
0	1	1.2·10^−6^
1	0	9.5·10^−5^
1	1	2.5·10^−5^

The functional forms used in the model are defined with parameters *Ω*_1_ and *Ω*_2_. Namely, parameter *Ω*_1_ defines competitive (0) or noncompetitive (1) binding sites at promoter level. Parameter *Ω*_2_ defines Michaelian (0) or linear (1) protein degradation form.

**Fig. 17:**
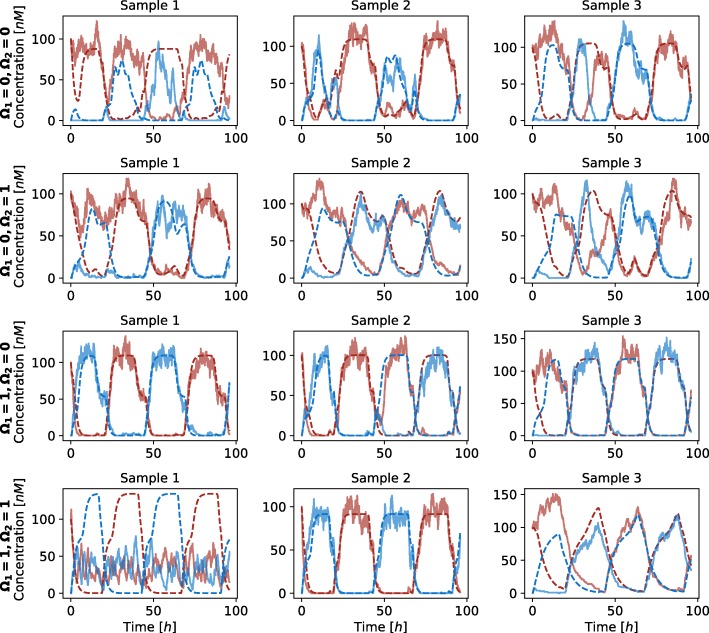
Comparison of stochastic and deterministic simulations performed on the D flip-flop model with different functional forms describing protein degradation and transcription factor binding at promoter level. *Ω*_1_ defines competitive (0) or noncompetitive (1) binding sites at promoter level, and *Ω*_2_ Michaelian (0) or linear (1) protein degradation form. Solid lines present the results of stochastic simulations and dashed lines present the results of deterministic simulations. Each of the lines presents the evolution of protein *q* (blue) and *q*_*c*_ (red) concentrations

**Fig. 18:**
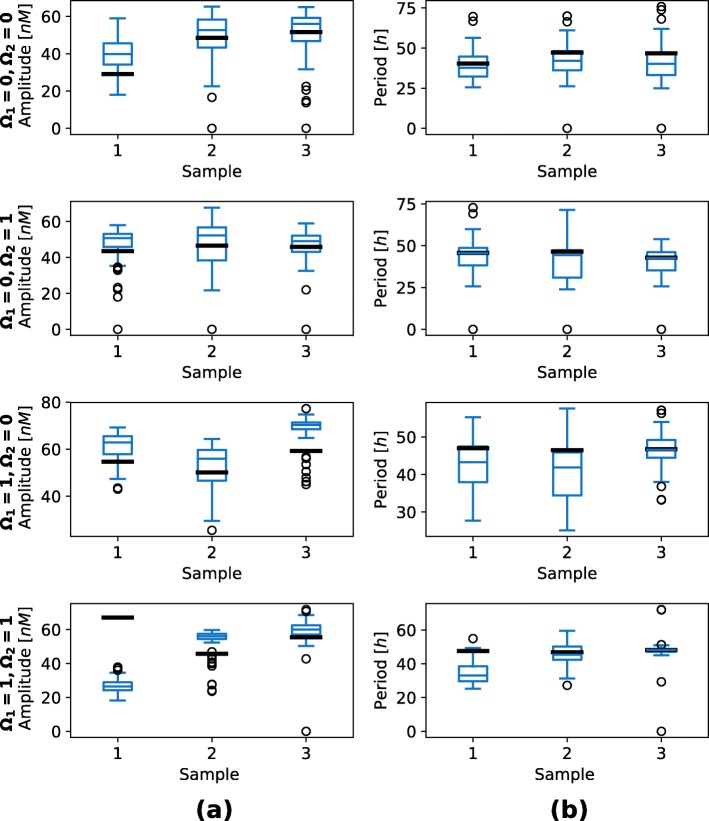
Boxplots visualizing the stochastic response of the D flip-flop model, namely amplitudes and periods of observed oscillations. *Ω*_1_ defines competitive (0) or noncompetitive (1) binding sites at promoter level, and *Ω*_2_ Michaelian (0) or linear (1) protein degradation form. Figures in column (**a**) present the average oscillation amplitudes and figures in column (**b**) the average oscillation periods. Sample numbers correspond to the numbers used in Fig. 17. A hundred simulations were performed for each sample. Average oscillation amplitudes and periods were measured in each of the simulations. Bold black lines present the results observed in deterministic simulations for the corresponding samples

We additionally validated the obtained results with the execution of stochastic simulations. We randomly selected three feasible random points for each functional form combination. Majority of the solutions pertained the response observed in deterministic simulations, however, the noise affected these solutions to a greater degree than in the repressilator or AC-DC circuit models (see Fig. 17). On the other hand, when execution large number of stochastic simulations, approximately the same response is observed as in deterministic simulations in all but one tested sample (see Fig. 18).

## Discussion

We developed the computational pipeline that can be used for *model-to-model* comparison in terms of the robustness to the perturbation of their kinetic parameters. Our work is based on the already established ’glocal’ method introduced by Hafner et al. [13]. In the first step, our approach roughly estimates global viable solution space through the optimization with the proposed genetic algorithm. Next, the viable solution space is more thoroughly explored with efficient local sampling. Because the viable solution space can be hyzz‘pothetically unconnected, we took a step further and proposed clustering to perform fine-grained exploration. The size and shape of viable regions can then be utilized for the assessment of model robustness. We successfully applied and validated our methodology on three distinct models that exhibit oscillatory and/or bistable behavior. Our approach utilizes exhaustive search of solution space, first by GAs and then with a prudent selection of samples, which is performed with local sampling in the direction of main principal components. We demonstrated the applicability of the proposed approach on three different deterministic ODE-based models.

One must also be aware of the potential drawbacks of the proposed approach. The first limitation is that the gap statistic and clustering are not perfect. There is not a strict consensus of what constitutes a separate cluster and different approaches will cluster the same population of samples differently. The drawback of gap statistic is that in order to test the null hypothesis, one must assume the distribution of the data points. We have shown, that if samples are uniformly distributed gap statistic always predicts the optimal number of clusters, however, this is not always the case for non-uniformly distributed data. To address this problem one could take methods for determining the number of clusters into account only partially as a guide and select the number of clusters based on observations. By our experience gap statistic consistently overestimated the number of clusters in the model of the AC-DC circuit and D flip-flop model. We approached this conservatively by setting the maximum number of clusters in the clustering step to 2 and by faster termination of the algorithm. The first viable region obtained by GAs can be clustered only once. All consecutive regions are then disregarded regardless of the gap statistic prediction.

The second possible drawback is that GAs and the probability based sampling introduces non-determinism into the sampling process, i.e. repetitive runs with the same configuration can return different results. We mitigated this with high population size and with a high probability of mutation and crossover in GA optimization. Similarly, we set the variance scaling factor *λ* relatively high at the beginning of our sampling process, when we were not as confident in the explored solution space, and gradually decreased it towards the end.

We have shown that the size and shape of a viable solution space are directly dependent on the definition of cost function and the threshold selection, which can be defined subjectively. We demonstrated the consequences of different interpretations of viable solution with the investigation of viable parameter regions on the same repressilator model for two different cost functions. The first cost function was defined in a strict manner, whereas the second one was defined more loosely. The viable parameter region of the repressilator model was greater when we employed the exploration of viable regions based on a loosely defined cost function. This coincides with our expectations and to some degree validates the correctness of our approach. In both cases, the parameter spans were roughly the same except for the degradation rate *δ*_*p*_ (see Fig. 6), which in the end contributed to the difference in the viable volume. Both cases have the same Hill coefficient ranges, 2≤*n*≤5. Higher values of Hill coefficient correspond to the positive cooperativity of transcription factors and a nonlinear response. The first implementation of a repressilator by Elowitz and Leibler [4] achieved non-linearity with the selection of transcription factors that function as oligomers, i.e. the TetR repressor protein is a dimer [33], and the LacI repressor is a tetramer [34].

To illustrate the exploration of viable regions with multiple well defined and unconnected clusters we analyzed the AC-DC model for two different modes of behavior. Our method proved to be effective in this scenario as well. Based on our observation, the AC-DC circuit tends more towards the oscillatory behavior than to bistability. We can assume this because the viable parameter volume is significantly larger for the parameter solutions that yield oscillatory dynamics in comparison to the bistable solutions. However, for the sake of simplicity, we excluded the signaling molecule *S* from the model and thus disregarded the switch like dynamics. It has already been shown that by adjusting the strength and/or the concentration of the signaling molecule the AC-DC circuit can exhibit both modes of behavior for the same sets of parameters [29]. Our simulations clearly indicate that in order to achieve either bistability or oscillations, the system should reflect non-linear transcription response. This can be described with larger values of the Hill coefficient [35]. Other researchers have already addressed this problem. For example, Lebar et al. [36] designed a bistable genetic switch based on designable DNA-binding domains of transcription-activator-like effectors (TALEs) [37]. Since TALEs bind non-cooperatively as monomers, a simple mutual repressor-based toggle switch does not support bistability. In order to introduce non-linearity and achieve bistability, Lebar et al. designed a bistable switch with an additional positive feedback loop of TALE repressors.

We investigated the robustness of the proposed D flip-flop model in dependence of different functional forms describing the protein degradation (Michaelian versus linear functions) and the transcription factor-promoter binding process (competitive versus independent binding). The flip-flop model proposed in [14] was extended into four different models upon which the proposed methodology was executed. We used the obtained results to assess the relative volumes of feasible solutions spaces in each of the models. These were used to perform the model-to-model comparison and to assess their robustness. We showed that Michaelian degradation form increases the chances of obtaining oscillations as already described in [38], but not as much as noncompetitive binding sites at promoter level [39]. When performing stochastic simulations we observed larger noise sensitivity as in the repressilator or AC-DC ciruit models. Solutions more resilient to intrinsic noise could potentially be obtained with the selection of different cost function defining feasibility of the solution, i.e. with larger oscillation amplitudes. The required amplitudes used in the D flip-flop model were set to 50 *nM* while amplitudes between 200 and 400 *nM* were regarded as feasible in the repressilator and AC-DC circuit models. Our results still indicate that the D flip-flop is robust to perturbations of its kinetic parameters and that the possibility of its implementation in the biological host is promising. Andrews et al. [30] demonstrated the feasibility of D flip-flop in *E. coli*. Still, their flip-flop is triggered on high signal levels and not on an edge of the synchronization signal. This makes the circuit hard to control, since the high level of the synchronization signal needs to be long enough to trigger the transition from one state to another and at the same time short enough to prevent multiple switches.

Our results indicate that the viable solution space of biological oscillators is generally well defined and connected, which has been already confirmed by other researchers [13, 17]. This is expected for the naturally occurring motifs that exhibit oscillations since they possibly evolved with random mutations that contributed to the small gradual changes of kinetic parameters [40]. However, it is interesting to observe similar properties in the synthetic circuits. This can be partially explained with the fact that the design is to some degree inspired by the systems we can observe in nature. Except for the AC-DC circuit, our models displayed single connected viable regions. The existence of multiple unconnected viable regions for the AC-DC circuit can be contributed to its capability of multimodal behavior. The values parameters span are generally different for both modes of behavior.

For a biological system to be robust, it must be able to withstand the fluctuations of biochemical parameters due to external factors, intrinsic noise, and single-cell variability. Our methodology can give one the insight into the shape and size of the viable parameter regions, and into the overall robustness of a system. Our approach has, therefore, two main applications. Firstly, by knowing the effect of parameters on system behavior, one could fine-tune the problematic parameters and use synthetic constructs, such as degradation tags to speed-up protein degradation [41], or design parts with higher binding affinities. For example, Fink et al. [7] designed coiled coils to increase the affinity between split proteases and thus increased the response of a system. However, approaches to experimentally tune the value of a given parameter are quite limited, especially in a predictive way. Secondly, one could use the proposed methodology to compare different systems with similar behavior and different topologies. For example, simple bistable switch could be compared with the bistable switch with positive feedback loops proposed by Lebar et al. [36]. The results of these comparisons could guide the researcher in the selection of more robust topologies and finally in the process of the implementation of reliable biological circuits.

We validated our methodology on biological GRN models that primarily exhibit oscillatory or bistable behavior. However, it is not hard to see how one could adapt this approach to cover other modes of behavior as well. By modifying the genetic algorithm and adjusting the cost function, one can adapt our approach to a variety of dynamical models and not only models of GRNs. We demonstrated the application of the proposed methodology solely on the results obtained with deterministic simulations. These models describe the average response of the system without the noise influences [42]. The noise influences can be to some degree indirectly analyzed with the analysis of parameter variability effects on the deterministic dynamics of the system [43]. In many cases it is more suitable to use stochastic modeling approaches, such as SSA [26]. These approaches directly describe the inherent stochasticity of biochemical reactions. In our case studies we only used the stochastic simulations to validate the correctness of the results obtained with deterministic approaches. However, these approaches could as well be used to generate the data upon which the proposed methodology would be applied. Moreover, the methodology could be used in a combination with any other either experimental or computational approach that is able to generate the response of the system at the sampled parameter values. However, there are some potential drawbacks in the straightforward application of these approaches. Deterministic models will always yield the same response for a given set of initial conditions and parameter values. Contrary, stochastic or experimental procedures will always differ to some degree even with the same conditions [27]. This means that multiple repetitions of the same experiment will be needed to achieve statistical significance, which in turn increases the time complexity of our methodology. Most of the approaches that present an alternative to deterministic models inherently increase the time complexity to generate the results even for a single simulation run. In order to at least partially circumvent this problem, one of the many parallelized adaptations of SSA with lower computational complexity could be applied [44].

## Conclusion

In this paper we proposed a novel approach that can be used to assess the viable parameter regions for an arbitrary GRN model, and which can be applied in the design of synthetic biological systems. Identified parameter regions allow us to compare different models in terms of their robustness and also to identify the critical segments of the selected system. This can be exploited for the design of more reliable and robust systems. For example, if the degradation rates of observed proteins are constrained to a small interval, one can then specifically focus on this segment by exploring the effects of different degradation tags [41] and thus fine-tune the problematic parameters. Moreover, our approach can be used as a foundation for other analyzes. For example, bifurcation or sensitivity analysis can be done more efficiently and with higher precision if one is confident in the size and shape of the viable solution space.

## Data Availability

All source code with accompanying results is available for download at: https://github.com/zigapusnik/AnalysisOfViableParameterRegions.

## Abbreviations

AC-DC: Alternative current-direct current
CLK: Clock
GA: Genetic algorithm
GRN: Gene regulatory network
MSE: Mean squared error
QSSA: Quasi steady-state approximation
SCSA: Structural and correlative sensitivity analysis
SSA: Stochastic simulation algorithm
TF: Transcription factor
