# Correction to: Computational analysis of viable parameter regions in models of synthetic biological systems

**DOI:** 10.1186/s13036-019-0213-0

**Published:** 2019-11-11

**Authors:** Žiga Pušnik, Miha Mraz, Nikolaj Zimic, Miha Moškon

**Affiliations:** University of Ljubljana, Faculty of Computer and Information Science, Večna pot 113, 1000 Ljubljana, Slovenia

**Correction to: J Biol Eng (2019) 13:75**

**https://doi.org/10.1186/s13036-019-0205-0**

The original version of this article [1] unfortunately included a typographical error to author Žiga Pušnik’s name.

The name was inadvertently spelled with a small instead of a capital letter. The correct name should be capitalized.

This has been included in the author list of this Correction article and is already updated in the original article.
